# Dynamics of epidemic diseases on a growing adaptive network

**DOI:** 10.1038/srep42352

**Published:** 2017-02-10

**Authors:** Güven Demirel, Edmund Barter, Thilo Gross

**Affiliations:** 1 Management Science & Entrepreneurship, Essex Business School, University of Essex, Southend-on-Sea, UK; 2 Department of Engineering Mathematics, University of Bristol, Bristol, UK

## Abstract

The study of epidemics on static networks has revealed important effects on disease prevalence of network topological features such as the variance of the degree distribution, i.e. the distribution of the number of neighbors of nodes, and the maximum degree. Here, we analyze an adaptive network where the degree distribution is not independent of epidemics but is shaped through disease-induced dynamics and mortality in a complex interplay. We study the dynamics of a network that grows according to a preferential attachment rule, while nodes are simultaneously removed from the network due to disease-induced mortality. We investigate the prevalence of the disease using individual-based simulations and a heterogeneous node approximation. Our results suggest that in this system in the thermodynamic limit no epidemic thresholds exist, while the interplay between network growth and epidemic spreading leads to exponential networks for any finite rate of infectiousness when the disease persists.

Throughout human history epidemic diseases have been a constant threat. The plague of Athens killed 25–35 percent of the city’s population as early as 430 BC^1^. A bubonic plague epidemic killed between 75000 and 100000 inhabitants of London between April 1665 and January 1666, when its population was about 460000^1^. Smallpox became a major threat to Europe throughout the eighteenth century, where growing cities like London were especially vulnerable to the disease on account of continuously providing immigrants that are susceptible to the virus^1^. After a brief respite during the 20th century epidemics are on the rise again. For instance, around 655000 people died of malaria in 2010^2^ and more than 100000 confirmed cases of Zika infection have occurred in the Americas in the past year^3^. Combating such epidemic diseases efficiently in the mega-cities of the future is likely to require a heightened understanding of the dynamics of the diseases and the social networks on which they spread.

In the past two decades, epidemic spreading has been extensively studied on different complex networks to understand the influence of the social contact network structure on the disease prevalence^4^,^5^,^6^,^7^. Two commonly studied models of infectious diseases are Susceptible-Infected-Susceptible (SIS) and Susceptible-Infected-Removed (SIR) models.

For SIS diseases on complex networks, the crucial determinant of epidemic spreading is the average maximum degree or the degree cutoff of the contact network where the hub node with the maximum degree and its neighbors act as an active incessant source for the endemic state^8^,^9^. Therefore, the epidemic threshold vanishes in the thermodynamic limit in networks where the maximum degree increases with the system size, which is true for most random networks unless there is a natural restriction on degree, for instance, due to cognitive limitations^7^.

For SIR diseases on complex networks, differently from SIS diseases, the fate of the disease is determined by the *degree distribution*, i.e. the probability distribution of finding an individual with a given number of contacts^8^,^10^,^11^. If the variance of this distribution is finite, then there is generally a threshold of infectiousness, which the disease has to exceed to reach a finite fraction of the population. By contrast, if the variance of the degree distribution is infinite, such as in certain scale-free networks, then the epidemic threshold vanishes and the SIR disease percolates on the network for all non-zero infectiousness levels via hierarchical spreading from hub nodes to lower degree nodes^12^. Thus, scale-free networks allow unlikely diseases with low infectiousness to spread and become endemic.

Today, the structure of social networks is recognized as a key factor with direct implications for epidemic dynamics and potential counter measures^13^,^14^,^15^,^16^,^17^. This insight has motivated the integration of real world network data into epidemic models^18^,^19^,^20^,^21^,^22^,^23^. Furthermore, theoretical models have been extended by including several properties of real-world networks such as degree constraints^24^, degree correlations^25^, clustering^26^,^27^,^28^, information filtering^29^, social hierarchy^30^, and nonuniform transmission probabilities^31^.

A relatively recent addition is to consider also the feedback of the epidemic on the social network structure, which can be indirect, e.g. by triggering behavioral changes of agents^6^,^32^, or direct by removing agents due to hospitalization, quarantine or death.

Modeling the network response to an ongoing epidemic leads to an adaptive network, a system with interplay between the dynamics of the network and the dynamics on the network takes place^33^,^34^. Epidemics on adaptive networks can exhibit complex emergent dynamics^32^,^35^,^36^,^37^,^38^ (e.g. sustained oscillations, bistability, and hysteresis) and emergent topological properties^32^,^35^,^39^,^40^ (e.g. heterogeneous degree distributions and assortative degree correlations). Furthermore, the study of the social response to epidemics is interesting from an applied point of view because it could enable enhanced vaccine control^41^ and effective quarantine strategies^42^.

In comparison to social responses to epidemics^32^,^35^,^36^,^37^,^38^,^39^,^43^,^44^,^45^,^46^,^47^,^48^, network growth and direct topological feedback via the removal of nodes have received less attention. Previous works considered the case where network growth and death processes are balanced and thus the population stays in equilibrium and fluctuates around a fixed system size^49^,^50^. The case of continuous growth was studied in Ref. 51, where new nodes attach preferentially to high degree non-infected nodes. It was observed that a transition from a scale-free topology to an exponential one takes place as the infectiousness decreases. Reference 52 study the effects of network growth and demographics on the dynamics of an SIS disease simultaneously spreading on the network, showing that the epidemic threshold vanishes in the thermodynamic limit. In another study, network growth and node removals have also been incorporated in a single model^53^. However, the authors focused on epidemic oscillations and did not consider topological effects in detail. Another related work focused on the interplay between network growth and dynamical behavior in the context of evolutionary game theory^54^, where new players preferentially attach to those receiving higher payoffs.

Here, we consider the growth of a network by preferential attachment from which nodes are simultaneously removed due to an SIR epidemic. The appeal of this model lies in its paradoxical nature, in absence of the disease, preferential attachment leads to the formation of scale-free topologies in which the epidemic threshold vanishes, such that the disease can invade. However, an established SIR disease will quickly infect and remove nodes of high degree such that the variance of the degree distribution is decreased and epidemic thresholds reappear, potentially leading to the extinction of the disease.

Now, consider the following line of reasoning: Observing a scale-free topology implies that the epidemic is extinct. But an extinct epidemic implies scale-free structure and hence vanishing epidemic threshold precluding extinction. Logically, the only possible solution is that the epidemic persists (unconditionally) in a network that is not scale-free. In other words, one would expect that the coevolution of epidemic state and network structure should lead to a vanishing epidemic threshold in an exponential network.

The argument presented above is admittedly hand-wavy. One objection against this line of reasoning that comes to mind immediately is that the paradox can also be resolved temporally, such that the epidemic goes extinct while the network is exponential. A disease-free scale-free network can then develop later since the disease cannot be reintroduced since no infected agents are left. However, this temporal resolution is only feasible in finite networks. In the thermodynamic limit it can be easily shown that a finite number of infected survive even below the epidemic threshold, which precludes complete extinction.

In the remainder of this paper we present a detailed dynamical analysis of the epidemic model using a heterogeneous node approximation along with detailed numerical computations. We show that disease-induced mortality reduces the variance of the degree distribution to a finite value, but not sufficiently far to cause the extinction of the epidemic. Thus, a balance is reached where the finite variance of the degree distribution is matched to the infectiousness of the pathogen. Therefore, the epidemic threshold vanishes in the thermodynamic limit, confirming the hand-waving argumentation above. We also identify a parameter region, where, after all, a temporal resolution of the paradox is observed.

## Methods

In this section, we first introduce the model and then develop our theoretical approach that describes the evolution of the network dynamics based on coarse-graining approximations.

### Model

We study the spreading of a susceptible-infected-removed (SIR) disease^55^ on an evolving network. In this network, a given node is either susceptible (state S) or already infected with the disease (state I). We start with a fully connected network of *m*_0_ nodes and consider three dynamical processes: a) the arrival of nodes, b) disease transmission, and c) the removal of nodes.

In the following we measure all rates per capita, including the arrival rate. This implies that larger populations have a proportionally larger influx of individuals, which appears plausible e.g. for growing cities, where the attractivity of the city increases with size. It is analogous to the use of per capita birth rates in models of population dynamics. We note that this assumption is necessary to keep the model well-defined in the thermodynamic limit.

New nodes arrive in the population at a constant per capita rate *q* and are already infected with the disease with probability *w*. Arriving nodes immediately establish links with *m* of the nodes selected according to the preferential attachment rule^56^: A new node establishes a link with a particular node *i* of degree *k*_*i*_ with a probability proportional to *k*_*i*_/∑_*j*_*k*_*j*_. Therefore, an incoming node’s links will attach to a node of degree *k* with probability *kp*_*k*_/〈*k*〉, where *p*_*k*_ denotes the degree distribution (the probability that a randomly picked node has degree *k*) and 〈*k*〉 = ∑ *kp*_*k*_ is the mean degree.

Disease transmission occurs at rate *p* on every link connecting a susceptible and an infected node. Therefore, nodes with higher degree are proportionally more likely to catch and spread the disease.

Removal of infected nodes takes place at rate *r*. Because we describe a fatal disease from which recovery is not possible, removed nodes and their links are entirely deleted and do not re-appear at a later stage. The removal of links along with the nodes depicts the rapid removal of corpses in the human population. The removal mechanism that is used here can also be considered as an approximate description for the hospitalization of infected individuals, which effectively removes the links. Furthermore, diseases in which dead hosts continue transmitting the disease can be captured in the same framework by a reduced removal rate *r*, taking into account the finite time between the death and the actual removal of the body.

Unless mentioned otherwise, the following set of parameter values is used throughout the paper: *m*_0_ = 6, *m* = 5, *q* = 0.01. In agent-based simulations the network is simulated until *N* reaches 10^7^ or the time reaches 10^4^.

### Analytical treatment

The dynamics on and of complex networks can be captured by a set of coupled ordinary differential equations, in so-called coarse-graining or moment-closure approximations^7^,^39^,^57^,^58^,^59^,^60^,^61^,^62^,^63^,^64^. In the next section we develop a heterogeneous node approximation, also called heterogeneous mean-field or degree-based mean-field approximation^7^,^11^,^65^,^66^, where the network evolution is captured in a set of equations for the node densities in different degree-classes.

Since the heterogeneous node approximation is in the form of a high-dimensional system of ordinary differential equations, we follow two directions for reducing the dimensionality of analysis. First we apply the mathematical triple jump approach^67^ to transform the infinite dimensional ordinary differential equation system in the thermodynamic limit to a two-dimensional partial differential equation system. Second we develop an alternative approach that reduces the heterogeneous node approximation to a low-dimensional ordinary differential equation system assuming random graph properties, which is later in the paper shown to be capable of estimating the network dynamics to high accuracy when away from the epidemic threshold.

#### Heterogeneous approximation

The heterogeneous node approximation consists of a set of ordinary differential equations for the densities [*A*_*k*_], the abundance of nodes in the class *A*_*k*_, which is the set of nodes of state *A* ∈ {*S, I*} and degree k, normalized by the total number of nodes *N*. The total density of S-nodes is denoted as [*S*], [*S*] = ∑_*k*_[*S*_*k*_]. The density [*I*] is defined analogously such that [*S*] + [*I*] = 1.

Before deriving the full equations for the specific system under investigation, we first illustrate the general structure of the heterogeneous moment expansion


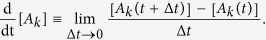


Here, two types of terms contribute to *d*[*A*_*k*_]/*dt*: a) changes in the abundance of nodes in the class *A*_*k*_, and b) changes in the normalization factor *N*.

For illustration, let us consider the process that removes infected individuals at a per capita rate *r*. For the moment we assume that these removals do not change N, as we treat the change in N separately below. When an infected node of degree *k* is removed, the abundance of nodes of type *I*_*k*_, *N*_(*I*,*k*)_, decreases by 1 so that the density [*I*_*k*_] is reduced by 1/*N*. Considering that (Δ*t*)*rN*_(*I*,*k*)_(*t*) such removal events take place within a time step Δ*t*, we obtain the rate of change for the density [*I*_*k*_] due to process a):


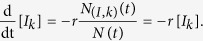


We now express the effect of the modification of the normalization factor *N* due to such removal events. When an infected node of arbitrary degree *k*′ is removed, the densities of all degree classes *A*_*k*_ are affected due to the modified normalization factor. In total, (Δ*t*)*rN*_*I*_ removal events take place within a time step Δ*t* resulting in [*A*_*k*_(*t* + Δ*t*)] = [*A*_*k*_(*t*)]*N*(*t*)/(*N*(*t*)  − 1), when isolated from the other changes of type a). Therefore, the rate of change of type b) due to removal events is


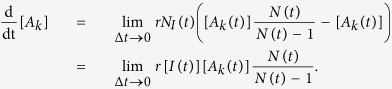


Noting that every node is updated on average once in a unit time, i.e. Δ*t* = 1/*N*, and taking the thermodynamic limit, we obtain the renormalization rate


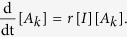


Writing the complete set of changes caused by infection, node arrival, and node removal processes, we derive the moment expansion for the densities [*S*_*k*_] and [*I*_*k*_]


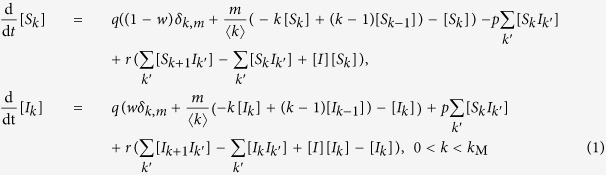


here, the quantity [*A*_*k*_*B*_*k*′_] denotes the density of links between nodes of type *A*_*k*_ and *B*_*k*′_, where *A, B* ∈ {*S, I*}, and *k* and *k*′ are the respective degrees.

For understanding the equation governing the evolution of the density [*S*_*k*_], consider that new nodes with degree *m* arrive at rate *q* and have state S with probability 1−*w*. Thus, the density [*S*_*m*_] increases at rate *q*(1−*w*). A newly arriving node builds a link to a node in the *S*_*k*_ class with probability *k*[*S*_*k*_]/〈*k*〉 and causes it to pass into the *S*_*k*+1_ class. Because *m* such links are established by each newly arriving node, the density [*S*_*k*_] decreases by *qmk*[*S*_*k*_]/〈*k*〉. Similarly, nodes in the *S*_*k*−1_ class pass into the *S*_*k*_ class at rate *qm*(*k*−1)[*S*_*k*−1_]/〈*k*〉. Additionally, as explained above, we need to renormalize the density [*S*_*k*_] when a node arrives. This corresponds to a loss of the [*S*_*k*_] density of *q*[*S*_*k*_].

At rate *p*, nodes within the *S*_*k*_ class become infected through their links with infected nodes of arbitrary degree *k*′, causing them to pass into the *I*_*k*_ class. The total density of such links is ∑_*k*′_[*S*_*k*_*I*_*k*′_].

Finally, nodes within the *S*_*k*_ class pass into the *S*_*k*−1_ class due to the removal of their infected neighbors of arbitrary degree *k*′. Given the density of such links, the density of [*S*_*k*_] decreases by *r*∑_*k*′_[*S*_*k*_*I*_*k*′_]. Similarly, nodes in the *S*_*k*+1_ class pass into the *S*_*k*_ class corresponding to a gain of *r*∑_*k*′_[*S*_*k*+1_*I*_*k*′_]. As infected nodes are removed, the density of all degree classes increases due to the renormalization leading to a gain of *r*[*I*][*S*_*k*_] for the density [*S*_*k*_]. The rate equation for the density [*I*_*k*_] is constructed analogously, with the addition of a term for the removal of infected nodes with degree *k* at the rate *r, r*[*I*_*k*_].

As the equations for node densities [*A*_*k*_] depend on link densities [*A*_*k*_*B*_*k*′_], Eq. (1) is not closed. In order to close the system, the moment expansion should be truncated by the moment-closure approximation, in which the densities of larger subgraphs are estimated in terms of the densities of smaller ones. Here, we use the heterogeneous node approximation to close the system at the node level


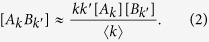


We have assumed that the nodes with the same degree can be considered identical and state and degree correlations between neighboring nodes are negligible. The mixing assumption generally requires a mixing or annealing process that makes it possible to replace the adjacency matrix structure with the degree distribution^8^, which is provided here by the constant removal and addition of nodes and links.

Using the node approximation of Eq. (2), we reach


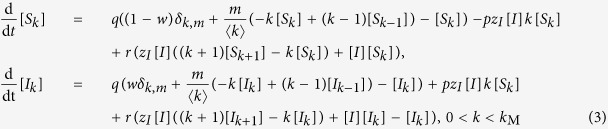


where *z*_*I*_ = 〈*k*_*I*_〉/〈*k*〉 and 〈*k*_*I*_〉 is the mean degree of infected nodes.

In the following we refer to Eq. (3) as the heterogeneous approximation. The main drawback of such heterogeneous approximations is the high dimensionality of the system of equations, which complicates the analytical solution and thus typically necessitates extensive numerical studies except for the analysis of special conditions. Furthermore, since it is not possible to numerically integrate an infinite dimensional system of differential equations, we need to introduce a degree cut-off *k*_M_ by assuming 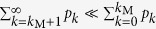. The higher the degree cut-off, *k*_M_, the more precise the heterogeneous approximation becomes.

#### Mathematical triple jump approach

The mathematical triple jump approach of ref. 67 consists of three steps. First, a high-dimensional ordinary differential equation system is developed to capture the dynamics under the types of heterogeneity that are identified to be of utmost importance. Second, the obtained system of ordinary differential equations is transformed to a low-dimensional partial differential equation system in the thermodynamic limit using moment generating functions. Finally, the partial differential equation system is analyzed using the tools of dynamical systems theory.

The first step has already been completed to find the heterogeneous approximation in Eq. (3). The second step is done below in this section, while the last step is carried out in the next section.

We first introduce the quantities *Q*(*t, x*) = ∑_*k*_[*S*_*k*_(*t*)]*x*^*k*^ and *R*(*t, x*) = ∑_*k*_[*I*_*k*_(*t*)]*x*^*k*^, which are the generating functions of the degree distributions of susceptible and infected populations. The time derivatives of *Q*(*t, x*) and *R*(*t, x*) are given by


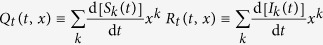


The partial derivatives with respect to *x* are defined analogously by


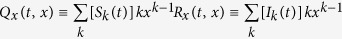


The functions *Q*(*t, x*) and *R*(*t, x*) are particularly useful because they are related to the moments as given below:


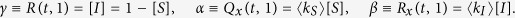


Using these quantities, we obtain the partial differential equations


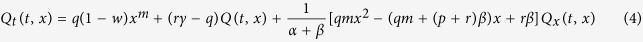



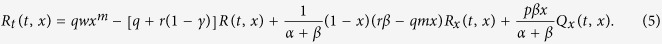


#### Homogeneous approximation

As a second alternative approach we develop a low-dimesional approximation by summing over the degree classes in Eq. (3). We consider the susceptible proportion of the population [*S*], the mean degree 〈*k*〉, and the mean degree of susceptibles 〈*k*_*S*_〉 which evolve according to


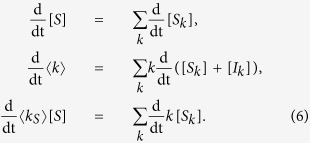


Using Eqs (3) and (6), we obtain


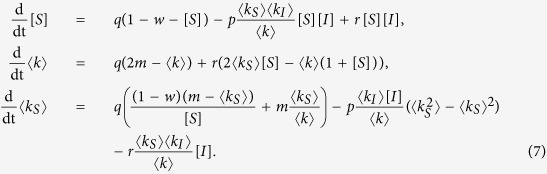


In the following, we refer to Eq. (7) as the coarse-grained heterogeneous approximation, which does not involve any further approximations other than those discussed above.

Because we have not derived an equation for the second moment of the susceptible degree distribution 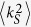, Eq. (7) does not constitute a closed dynamical system. We address this problem by replacing 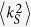 by 〈*k*_*S*_〉^2^ + 〈*k*_*S*_〉 in an additional approximation. We note that this approximation is valid exactly when the network has a Poisson degree distribution. It can therefore be thought of as a ‘random-graph approximation’. This approximation will certainly fail in the case of scale-free networks because of the degree distribution’s diverging variance, i.e. 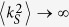, in which case we will resort to the heterogeneous approximation and its PDE description in the thermodynamic limit for the analysis of the model. However, as will become apparent below, the system obtained by the random-graph approximation still performs well for distributions with large finite variance.

Using the random-graph approximation we obtain


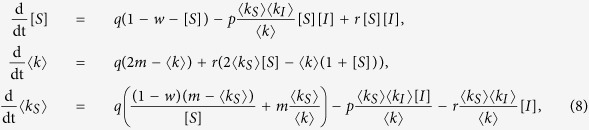


where [*I*] and 〈*k*_*I*_〉 are given by the equations [*S*] + [*I*] = 1 and 〈*k*_*S*_〉[*S*] + 〈*k*_*I*_〉[*I*] = 〈*k*〉, such that the system constitutes a closed model. In the following we refer to this model as the homogeneous approximation.

## Results

In the previous section, we first present the analysis of heterogeneous and homogeneous approximations and confirm the results by comparison with individual-based simulations of the network. Later we present a detailed analysis of the epidemic threshold. We finally discuss the emergence of dynamics that involve epidemic cycles.

### General properties of the network and disease prevalence

Before we launch into a detailed discussion of the model, let us consider the limiting case of network evolution in the absence of the epidemic. In this case the model is identical to the Barabási-Albert model of network growth^56^, which is known to lead to scale-free topologies, where the degree distribution follows a power law *p*_*k*_ ∝ *k*^−*γ*^ with exponent *γ* = 3 and thus the degree variance *σ*^2^ diverges in the disease-free state. Because the density of infected vanishes in the absence of the epidemic, it is also evident that the degree distribution must be scale-free independent of the parameters *p* and *r*.

In the present model the emergence of scale-free topologies is thus expected in the limit where the disease goes extinct or remains limited to a finite number of infected nodes 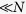. When the epidemic is present, high degree nodes are disproportionately likely to become infected and subsequently removed, which can be expected to prevent the formation of scale-free topologies.

**Figure 1:**
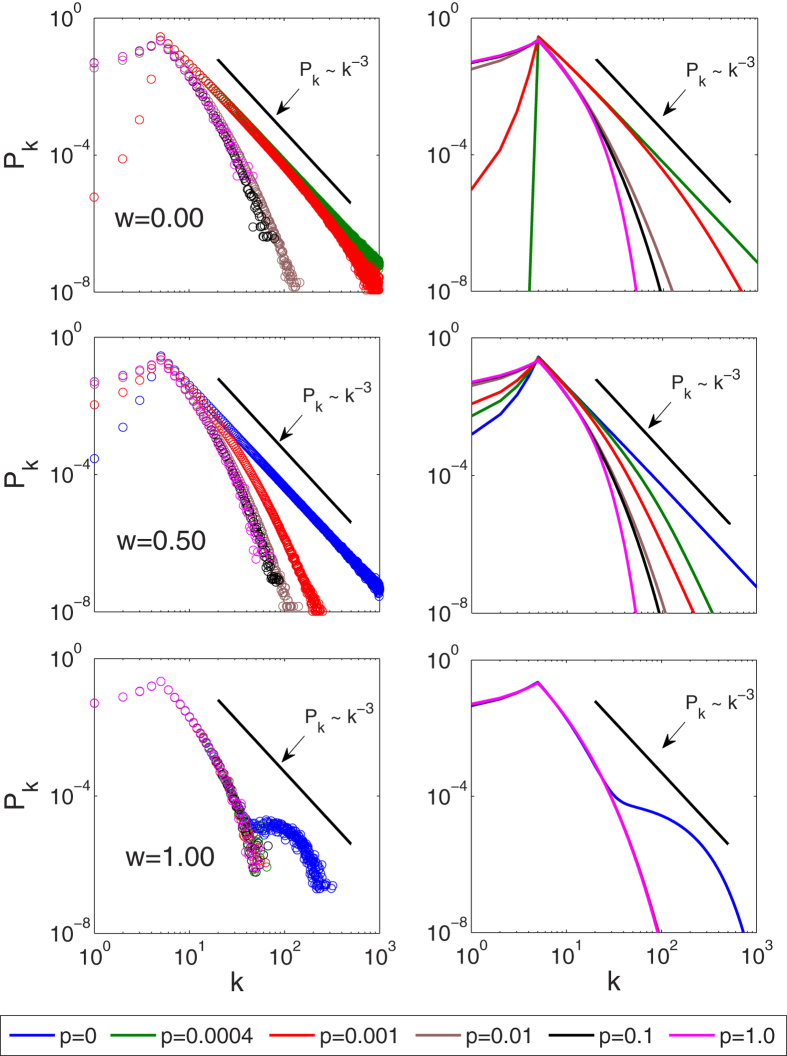
Degree distribution for varying infectiousness *p* and infected arrival fraction *w*. Left: individual-based simulations. Right: heterogeneous approximation. Scale-free degree distributions are observed when disease infectiousness vanishes (*p* = 0). For increasing infectiousness the degree distribution quickly becomes exponential. Parameters: *r* = *q* = 0.01, *m*_0_ = 6, *m* = 5. Shown are averages over 1000 simulation runs.

We confirm this intuition by plotting degree distributions for various parameter sets in Fig. 1. We show a comparison of the heterogeneous approximation with individual-based simulations. The figure shows a good agreement between the modeling approaches and confirms basic intuition. When all arriving nodes are susceptible (*w* = 0), a scale-free degree distribution with the expected exponent *γ* = 3 is formed for *p* = 0. At finite infectiousness *p*, the topology changes from scale-free to exponential. The same behavior is observed at higher rates of infected arrivals, 0 < *w* < 1.

When all arriving nodes are already infected (*w* = 1), the distribution has a bimodal form for *p* = 0 with high degree contribution coming from the initial susceptibles which never get infected. At positive infectiousness *p*, these individuals eventually die and the mode at high degrees disappears.

**Figure 2:**
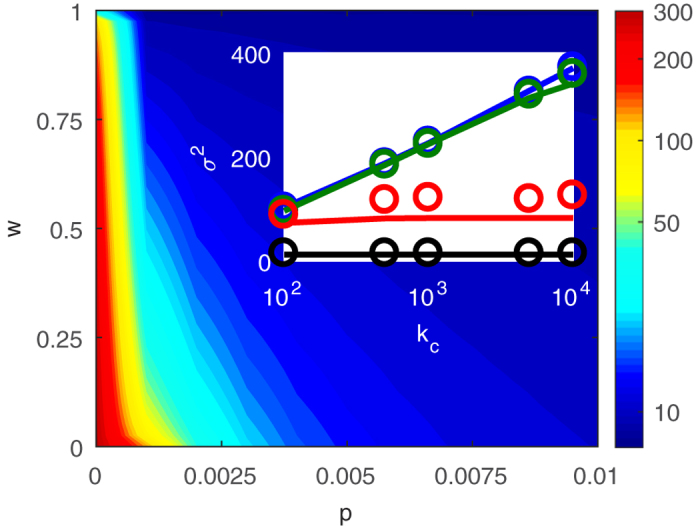
Variance σ^2^ of the degree distribution. Degree variance σ^2^ is plotted as a function of the infectiousness p and the infected arrival fraction *w* from agent-based simulations. The degree variance *σ*^2^ decreases with increasing infectiousness *p* and fraction of infected arrivals *w*. Inset: Degree variance *σ*^2^ as a function of the degree cut-off *k*_*c*_. A transition from a cut-off dependent (*p* = 0, scale-free) to an independent (*p* = 0.0004, *p* = 0.001, and *p* = 0.005, exponential) regime is observed as the infectiousness *p* is increased. In network simulations (circles), nodes were restricted to at most *k*_*c*_ neighbors. In the heterogeneous approximation (solid lines) the cut-off *k*_*c*_ is directly imposed as *k*_M_. Parameters: *r* = *q* = 0.01, *m*_0_ = 6, *m* = 5, *k*_*c*_ = 5 × 10^3^.

In order to quantify the topological transition from the scale-free to the exponential degree distribution, we plot the variance *σ*^2^ of the degree distribution as a function of infectiousness *p* and fraction of infected arrivals *w* in Fig. 2. As either parameter increases, the disease prevalence in the steady-state, [*I*]^*^, increases and removal occurs at a high rate. As a result the degree distribution becomes narrower and the degree variance decreases. It is apparent that very high values of the variance are only found for low infectiousness *p*, whereas higher infectiousness quickly leads to narrow distributions.

Above we computed the variance *σ*^2^ of the degree distribution of networks. One concern in any computation of this kind is finite-size effects. In the heterogeneous approximation these effects appear directly in form of the maximal degree that is considered in the approximation. In agent-based simulation a similar cut-off exists as the maximal degree in a network of finite size is bounded by the number of nodes. Hence all moments of the degree distribution, including the variance, must be finite regardless of the shape of the degree distribution. However, if the finite networks are drawn from an ensemble that becomes scale-free in the thermodynamic limit the variance *σ*^2^ is often found to increase logarithmically with the imposed cut-off 24.

We now rule out that low values of the degree variance *σ*^2^, observed above, were due to finite-size effects by considering the variance *σ*^2^ as a function of the degree cut-off *k*_*c*_ (Fig. 2 inset). For the case of *p* = 0, where we observed scale-free behavior, we find that the observed variance increases logarithmically as expected. Conversely, for the finite values of infectiousness *p* the observed *σ*^2^ is insensitive to a sufficiently large cut-off. In summary these results show that fatal diseases should relatively quickly destroy scale-free structure of social networks at all but the smallest removal rate and/or infectiousness.

**Figure 3:**
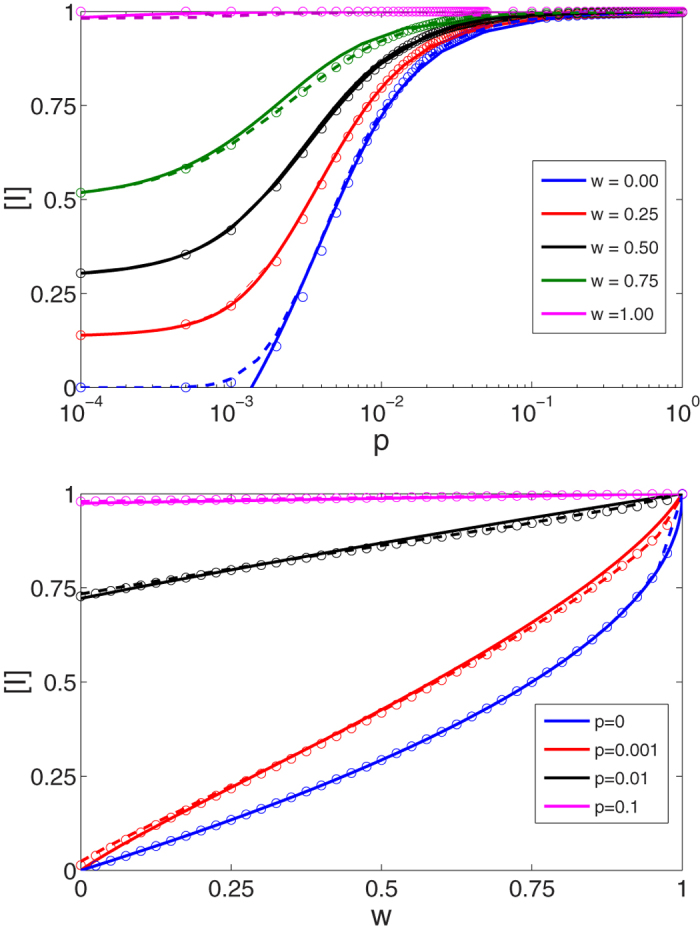
Dependence of the disease prevalence [*I*]^*^ on infectiousness *p* (top) and fraction of infected arrivals *w* (bottom). The disease prevalence increases monotonically with infectiousness *p* and fraction of infected arrivals *w*. In agent-based simulations (circles), [*I*]^*^ is calculated over the surviving runs among 10^3^ total realizations. Homogeneous approximation (solid lines) is the analytical solution of Eq. (8). Heterogeneous approximation (dashed lines) is the stationary value of the numerical integration of Eq. (3). Parameters: *r* = *q* = 0.01, *m*_0_ = 6, *m* = 5.

**Figure 4:**
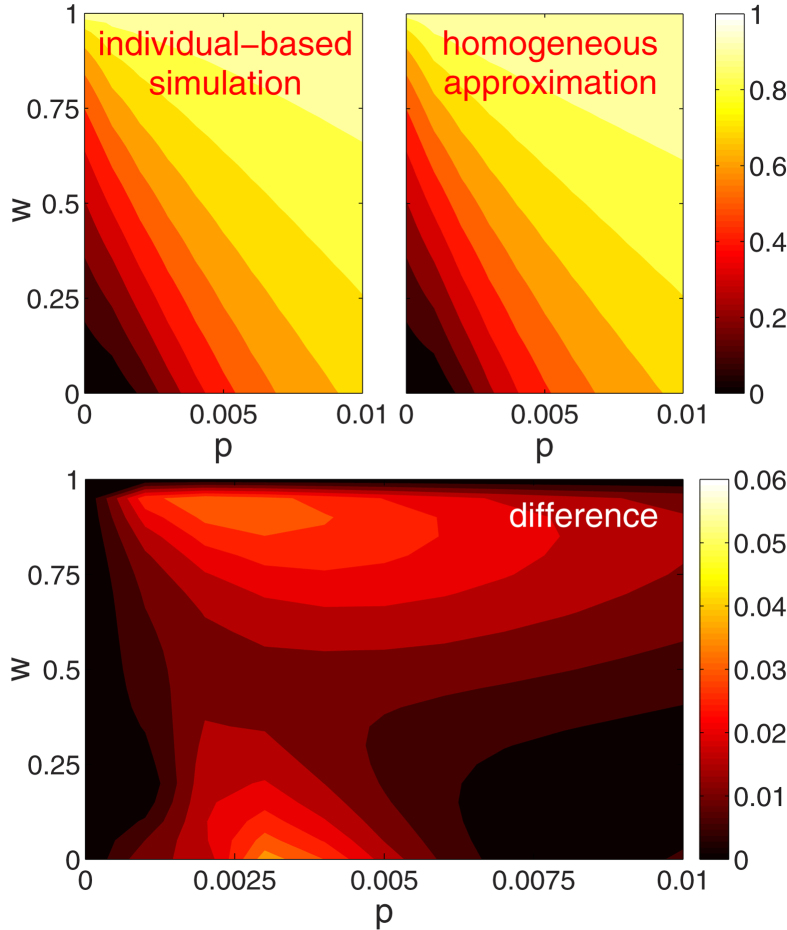
Performance of the homogeneous approximation in estimating the disease prevalence [*I*]^*^ in the (*p, w*) parameter space. Top-left: individual-based simulations. Top-right: homogeneous approximation. Bottom: absolute difference between individual-based simulations and the homogeneous approximation. Homogeneous approximation performs well for a large range of infectiousness *p* and infected arrival fractions *w*. Parameters: *r* = *q* = 0.01, *m*_0_ = 6,*m* = 5.

Let us now investigate the effect of the emergent network topology on the prevalence of the disease. Plots of the disease prevalence as a function of the infectiousness *p* and the fraction of infected arrivals *w* are shown in Figs 3 and 4. Figure 3 shows that the heterogeneous approximation (dashed lines) is in very good agreement with the agent-based model for the whole range of parameters *p* and *w*. The precision of the homogeneous approximation is also high for a large range of parameter values. As illustrated in Fig. 4, the absolute error in estimation of the disease prevalence of the approximation is maximal for intermediate values of infectiousness *p*, but still less than 0.05. The only qualitative discrepancy between the approximation and the agent-based model emerges at low infectiousness *p* for zero infected arrivals (*w* = 0). Here, the homogeneous model predicts the existence of an epidemic threshold, whereas in the agent-based simulation and the heterogeneous approximation the disease is found to persist even for very low levels of infectiousness *p*.

Summarizing the results shown so far, we can say that ongoing epidemic dynamics quickly leads to the formation of networks with finite variance. Generally,one would expect that such networks should exhibit a finite epidemic threshold. Nevertheless, the heterogeneous approximation and simulations indicate that the epidemic can persist in these networks for any finite positive value of the infectiousness.

### Analysis of the epidemic threshold

Here, we investigate in greater detail the apparent absence of the threshold, which is implied by the general analysis above. Throughout the argument we will only consider the case where all arriving agents are susceptible, *w* = 0.

The epidemic threshold is commonly defined as the minimal value of the infectiousness, below which a randomly picked node is susceptible with probability 1. In any finite network this implies that below the epidemic threshold each individual node is susceptible. By contrast in the thermodynamic limit there can still be a finite number of infected nodes as long as the density of such nodes in the network is zero, i.e. [*I*] = 0. In the following we denote the state below the epidemic threshold as the disease-free state, but recognize that there may be still a finite number of infected individuals.

We now calculate the epidemic threshold from Eq. (3) by looking at the stability of the disease-free state represented by [*I*] = 0 and hence [*I*_*k*_] = 0 for all *k*. The disease-free state is stable if all eigenvalues of the corresponding Jacobian matrix have negative real parts. The epidemic threshold is characterized by a bifurcation point where the leading eigenvalue of the Jacobian is zero. The disease-free state becomes unstable while a stationary state with non-zero disease prevalence becomes stable beyond the epidemic threshold.

The Jacobian matrix is obtained by the linearization of Eq. (3) around the steady state. We will shortly refer to the Jacobian matrix evaluated at the disease-free steady state as the Jacobian and denote it J = [*J*_*ij*_], where *i, j* = 0, 1, 2, …, *k*_M_. The Jacobian has a block matrix structure


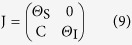


where Θ_S_ ≡ ∂_S_dS/dt, Θ_I_ ≡ ∂_I_dI/dt, and C ≡ ∂_I_dS/dt, all evaluated at the disease-free state and with the definitions 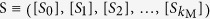 and 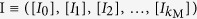.

The epidemic threshold is characterized by zero eigenvalue of the Jacobian, which is given by the solution of 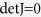. From Eq. (9), the determinant of the Jacobian can be obtained from the equality of 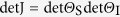.

Taking the partial derivatives of Eq. (3), we obtain


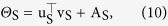


where u_S_ = [*u*_S_(*k*)], v_S_ = [*v*_*S*_(*k*)], and A_S_ = [A_S_(*j, k*)], for *j, k* = 1, 2, …, *k*_M_ + 1, with matrix entries


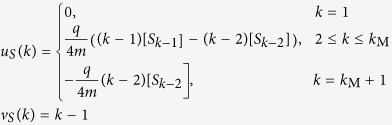



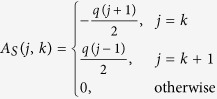


From the matrix determinant lemma, 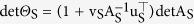.


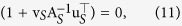


which can be computed exactly given that, for 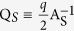 and Q_*S*_ = [*Q*_*S*_(*j, k*)], the entries of Q_*S*_ are given by the recursion equation


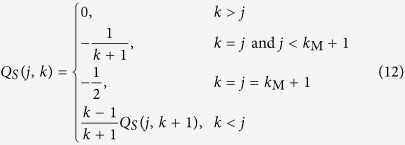


We note that the matrix Q_S_ provides also the densities [*S*_*k*_] in the disease-free steady state for finite degree cut-off *k*_M_. The vector of steady state susceptible densities, S, can be solved from


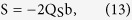


where


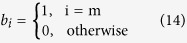


We have now established an explicit solution for 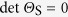 for any *k*_M_, which is obtained by inserting Eqs (12) and (13) into Eq. (11).

We now follow the same procedure to solve 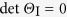, which constitutes the other possible solution of 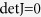. This solution is given by


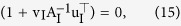


where


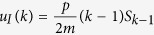



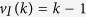



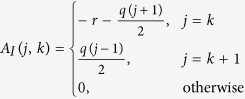


The matrix inverse 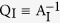 is solved from the recursive equation


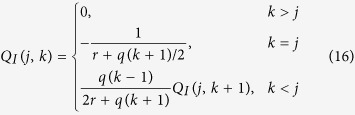


**Figure 5:**
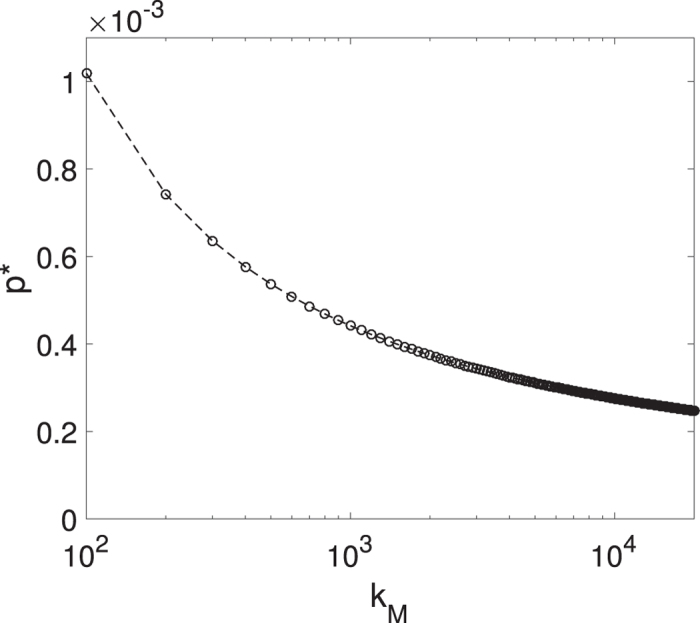
The epidemic threshold P^*^ calculated from Eq. (15) as a function of the degree cut-off *k*_M_. The epidemic threshold vanishes in the thermodynamic limit. Parameters: *r* = *q* = 0.01, *w* = 0, *m*_0_ = 6, *m* = 5.

The epidemic threshold needs to satisfy one of Eqs (11) or (15) for any finite degree cut-off *k*_M_. Our investigations show that Eq. (11) is ruled out and the epidemic threshold is given by Eq. (15). Figure 5 shows the epidemic threshold calculated from Eq. (15) for increasing values of degree cut-off *k*_M_, which approaches zero in the thermodynamic limit. This result is contraintuitive, as the epidemic threshold vanishes even in a system where the degree variance is finite.

For verification, we now take a complementary approach and calculate the epidemic threshold from Eqs (4) and (5). Setting time derivatives to 0 and *x* = 1 in both Eqs (4) or (5), we obtain


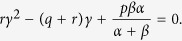


for the case *w* = 0. Using the definitions in Eq. (4) we find


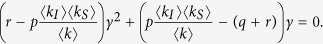


As expected there are two solutions for the steady state fraction of nodes infected, *γ*. Factoring out the uninfected state *γ* = 0 gives the non-trivial root


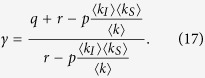


For this root to give a value of 0 ≤ *γ* ≤ 1 requires 
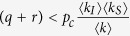
.

One can now ask if 
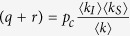
 constitutes a finite threshold below which the endemic state becomes unphysical. At such a threshold the number of infected nodes would become zero, leading to a double root at *γ* = 0. At this point 〈*k*〉 ≈ 〈*k*_*s*_〉 and therefore


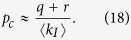


We note that for large networks the probability of a node being infected scales as *kp*_*k*_. Therefore in the case of the scale-free degree distribution, *p*_*k*_ ~ *k*^−3^, which is bound to form at the threshold, if one existed, the mean degree of infected nodes, 〈*k*_*I*_〉, is undefined. Hence in the limit of large networks *p*_*c*_ → 0 and the threshold vanishes.

Further, in the thermodynamic limit the network cannot reach a disease-free steady state. In the thermodynamic limit, when *γ* = 0 a finite number of nodes will be infected. In this situation preferential attachment leads to a degree distribution with infinite variance, as node removal is so small. At the moment the variance of the degree distribution becomes infinite, we anticipate 〈*k*_*I*_〉 to also become infinite and the limit *p*_*c*_ to go to zero. With the epidemic limit at zero the disease will be abel to spread in the network.

**Figure 6:**
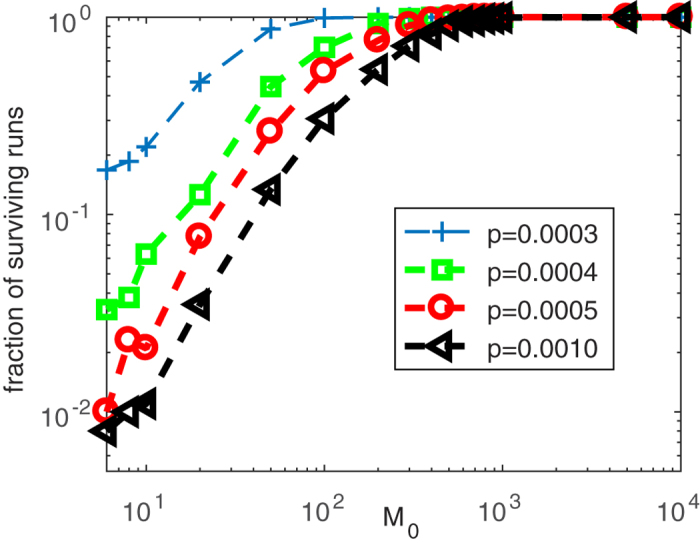
The fraction of surviving runs for low infectiousness *p* as a function of the initial network size *M*_0_ . The fraction of surviving runs increases as the initial network size *M*_0_ increases. In order to ensure the same initial average degree *m*, the Barabási-Albert growth model with *m* = 5 and *m*_0_ = 6 is iterated until the network reaches size *M*_0_, then the full model simulation with disease dynamics starts. Then nodes are assigned states and the infection, removal, and network growth processes take place simultaneously. Parameters: *r* = *q* = 0.01, *w* = 0, *m*_0_ = 6, *m* = 5.

We note that in finite networks the temporal dynamics can lead to a disease-free scale-free state. In this case the network goes through an initial exponential phase where an epidemic threshold exists that leads to the extinction of the epidemic. Subsequently, a scale-free topology develops, in which the epidemic threshold vanishes. However the epidemic cannot reappear as no infected are left which could reignite the epidemic. We emphasize that this mechanism is a finite-size effect. The finite-size extinction of the epidemic is demonstrated explicitly in Fig. 6, which shows that the fraction of individual-based simulation runs in which the epidemic persists increases with the initial network size *M*_0_. For populations that start with more than a few individuals the effect can be neglected and epidemics persist indefinitely.

### Further dynamics

Precluding the finite-size effect described above, simulation runs in the parameter range considered so far approach a finite prevalence for any positive value of infectiousness. The paradox outlined above is thus resolved by alternative a) mentioned in the introduction, that is the model does not have an epidemic threshold although the second moment of the degree distribution remains finite. As a final step in our exploration present some evidence that the temporal solution of the paradox, alternative b), is also possible.

**Figure 7:**
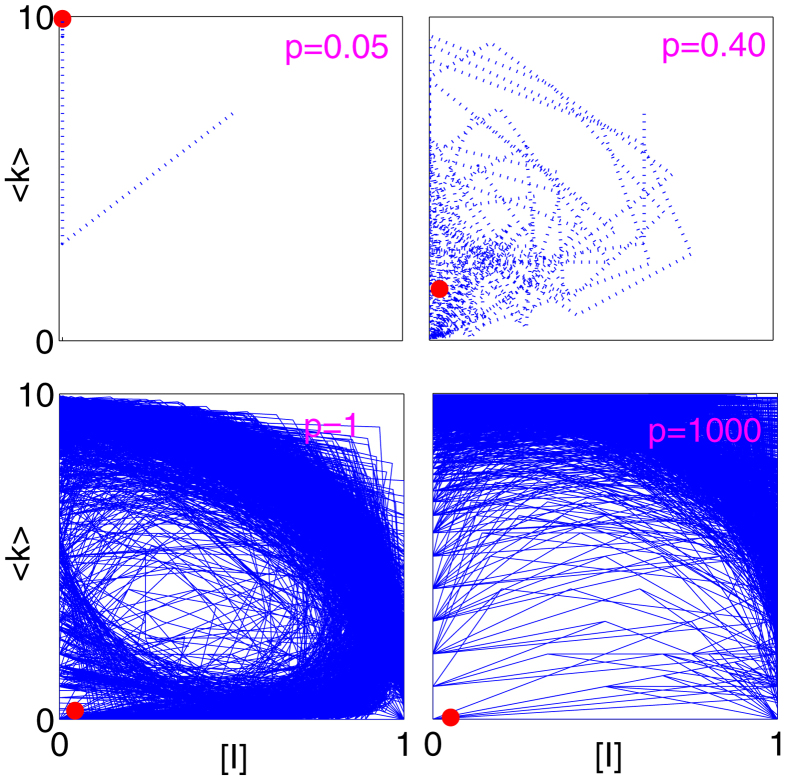
The dynamical evolution in the [*I*]−〈*k*〉 plane for high removal rate *r*. At high infectiousness *p*, the observed behavior resembles a homoclinic trajectory. The network grows from a healthy initial state until an infected individual eventually arrives and the disease quickly spreads over the network and the infected individuals subsequently die. Homogeneous approximation (red circles) fails to capture this behavior and predicts a stable equilibrium. Parameters: *q* = 0.01, *r* = 0.10, *m*_0_ = 6, *m* = 5, *w* = 0.1.

Up to now we have considered relatively small removal rates *r*. At sufficiently high removal rate, the evolution of the disease and the topology exhibits dynamics different from the convergence to a stationary state mainly due to the dominance of finite size effects. Figure 7 shows a representative evolution at non-zero fraction of infected arrivals *w*. At the combination of high infectiousness *p* and high removal rate *r*, the dynamics resembles a homoclinic trajectory in the 〈*k*〉−[*I*] plane. The disease spreads quickly over the network and covers the whole population immediately. Then disease-induced removals dominate and the population becomes extinct until new healthy individuals arrive and the disease spreading restarts, which leads to cycles of population growth and collapse.

When all arrivals are susceptible, i.e. *w* = 0, the epidemics in the finite population disappears entirely and the collapse-and-growth cycle cannot be completed. A disease-free scale-free network then emerges. Based on the arguments above one can suspect that the same behavior cannot occur in the thermodynamic limit. Instead it is likely that the observed dynamics forms part of a homoclinic cycle, where long phases of very low disease prevalence are disrupted by sharp outbreaks.

The observed dynamics at high removal rates would correspond to diseases with very high mortality where infected individuals die almost immediately. This is reminiscent of the massive pandemics in history, where humanity was exposed to new pathogens with very high virulence. While such diseases could lead to the deaths of large fractions of populations, continuous supply of healthy individuals through immigration provided new hosts to the disease and introduced new bursts of disease spreading which caused repeated epidemic cycles.

## Discussion

In the present paper we have investigated the dynamics of a fatal SIR disease in a growing population. Our main finding is that no epidemic threshold exists in this model. Although the variance of the degree distribution remains finite in the evolved topologies topologies “unlikely” diseases with very low infectiousness can persist indefinitely.

We presented a detailed analytical exploration for the case of low removal rate, where the prevalence of the disease reaches a stationary level. In the growing population the ongoing epidemic dynamics eliminates the nodes of high degree and thus leads to the formation of topologies for which the variance of the degree distribution is finite. However, this mechanism only reduces the width of the degree distribution so far that the epidemic can still persist. Therefore, the disease itself can not reduce the degree variance so much to cause its own extinction. For any finite value of the infectiousness the network adapts its topology such that the variance of the degree distribution is lowered to point where the epidemic can still be sustained, which explains the observed absence of the epidemic threshold.

When the removal rate is sufficiently high, network simulations indicate that the disease can no longer stabilize at a stationary level and instead growth-and-collapse cycles are observed. Such dynamics are reminiscent of homoclinic orbits, which have for instance been observed in an adaptive-network model of social cooperation^68^.

The dynamical feedback between the population structure and the epidemic disease has been so far studied in a number of articles in the past couple of years^32^,^35^,^36^,^37^,^38^,^39^,^43^,^44^,^46^,^47^. However, models captured mostly social interactions in non-fatal diseases. A significant obstacle to progress in this line of work if that the network evolution in most models is driven by behavioral changes of individuals (whom to meet, how often to wash hands). However, despite the ever increasing availability of data, for behavior often no records exist such that model predictions cannot easily be compared with real world data. By contrast, in the model proposed here, the social network evolves due to demographic processes such as migration and death on which data may be easier to obtain.

The model introduced here can be extended in several directions. For instance, dead hosts may not be immediately removed from the system and continue to spread the disease for a finite time until eventually being removed, the disease may not certainly lead to death such that infected individuals can recover, newly arriving individuals may establish links with only susceptible individuals, but not infected individuals, and susceptible individuals may rewire their links with infected neighbors to other susceptible individuals as a precaution against the epidemic. Independently of the specific mechanism, the disease-mortality combined with the network growth process can be expected to hold in all systems in which the dynamics of the population, in absence of the epidemic, leads to topologies with diverging variance of the degree distribution.

We believe that the proposed model is relevant for epidemics in rapidly growing cities, especially in the developing countries. In this context, connecting the model to real world data will be feasible in the future. In these situations we anticipate there is no epidemic threshold as the relevant populations often comprise many millions and are hence sufficiently large that the thermodynamic limit is a reasonable approximation, and the arrival of infected immigrants reintroduces diseases to the population. One can easily imagine extensions of the present model that incorporate policy measures such as vaccination, quarantine, or regulation of migration. We hope that this will in the future lead to the formulation of more efficient policies for combating epidemic diseases.

## Additional Information

**How to cite this article:** Demirel, G. *et al*. Dynamics of epidemic diseases on a growing adaptive network. *Sci. Rep.* **7**, 42352; doi: 10.1038/srep42352 (2017).

**Publisher's note:** Springer Nature remains neutral with regard to jurisdictional claims in published maps and institutional affiliations.
